# Public health round-up

**DOI:** 10.2471/BLT.20.010220

**Published:** 2020-02-01

**2020: Year of the Nurse and Midwife**

Mary Uwingabire, a midwife at Kawempe Referral Hospital, Uganda, holding the baby of Kabugho Moureen. Nine million more nurses and midwives are needed, if countries are to make progress towards universal health coverage. 2020 is the International Year of the Nurse and the Midwife, the theme of this year’s World Health Day on 7 April. http://bit.ly/2RiRzk1

**Figure:**
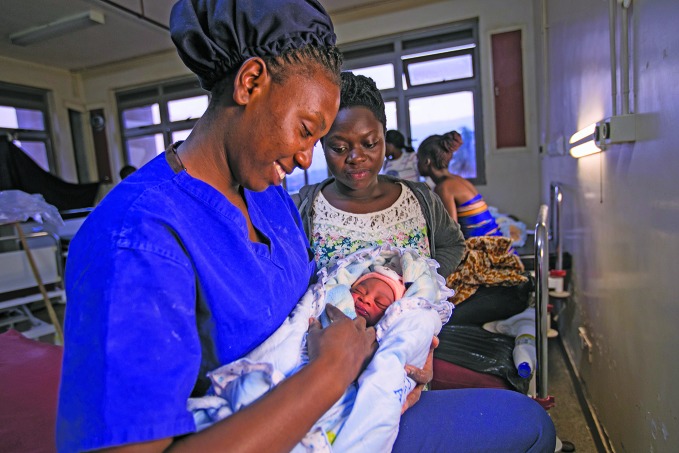
UNICEF/Zahara Abdul

## New coronavirus 

The World Health Organization (WHO) was working with officials in Thailand and China last month, following confirmation of an infection with the novel coronavirus (2019-nCoV) in a person in Thailand.

The person was a traveller from Wuhan, China and was identified by Thai officials on 8 January, and hospitalized that day. The person was recovering from the illness, according to Thai officials. 

This is the first exported case of novel coronavirus from Wuhan, China, as of 14 January, after China reported 41 cases with a preliminary diagnosis of 2019-nCoV infection, including 1 death in a person with an underlying medical condition.

Exported cases were expected and this first identified case reinforces WHO’s calls for active monitoring and preparedness in other countries. WHO has issued guidance on how to detect and treat infected persons.

The genetic sequencing data shared by China enable more countries to rapidly diagnose patients. WHO reiterated that investigations need to continue in China to identify the source of the outbreak and any animal reservoirs or intermediate hosts. 

http://bit.ly/2TB941Z

## Men’s tobacco epidemic turns corner

The number of men using tobacco is declining globally for the first time in spite of population growth, indicating a shift in the global tobacco epidemic, according to the *WHO global report on trends in prevalence of tobacco use 2000-2025* that was released in December.

The number of male tobacco users, which had previously been increasing, turned the corner in 2018 and is projected to decline each year from 2019, if tobacco control efforts are maintained.

Overall global tobacco use has fallen by about 60 million people, from 1.397 billion users in 2000 to 1.337 billion users in 2018.

This drop in tobacco use has been largely driven by reductions in women users: in 2018, 244 million women were using tobacco compared with 346 million women in 2000.

Over the same period, the number of male tobacco users increased by around 40 million, from 1.050 billion in 2000 to 1.093 billion in 2018, representing about 82% of the world’s current 1.337 billion tobacco users.

The new report shows, however, that the number of male tobacco users has stopped increasing and is projected to have declined by 2 million to 1.091 billion this year and by 5 million to 1.087 billion in 2025 as compared with the 2018 level.

By 2020, WHO projects there will be 10 million fewer tobacco users, male and female, compared to 2018, and another 27 million fewer by 2025. A total of 130 countries have been experiencing a decline in tobacco use since 2010. 

http://bit.ly/2t8AHod

## Measles in the Democratic Republic of the Congo

WHO called for more funding to stop the measles outbreak in the Democratic Republic of the Congo. As of 5 January, the health ministry had reported a cumulative total of 316 454 cases and 6102 deaths since the beginning of 2019.

During the first week of this year, 4983 new cases were reported and 57 deaths.

A vaccination campaign led by the health ministry reached more than 18 million children under five years of age across the country in 2019 with support from WHO, Gavi, the Vaccine Alliance, and other agencies.

However, in some areas, routine vaccination coverage remains low and about a quarter of the reported measles cases are in children over the age of five.

“We are doing our utmost to bring this epidemic under control. Yet, to be truly successful, we must ensure that no child faces the unnecessary risk of death from a disease that is easily preventable by a vaccine. We urge our donor partners to urgently step up their assistance,” said Dr Matshidiso Moeti, WHO Regional Director for Africa.

The epidemic has been fuelled by low vaccination coverage among vulnerable communities, malnutrition, weak public health systems and difficult access to health services. In addition, insecurity has also hampered the measles response in some areas.

Lack of funding is also a major barrier to ending the outbreak. So far, US$ 27.6 million has been mobilized but a further US$ 40 million is needed for a six-month plan to extend the vaccination to children between the age of six and 14 years, and to reinforce the outbreak response. 

http://bit.ly/3aeVS8u

## Obesity and undernutrition

Countries need to re-orient their food systems towards healthier nutrition to reduce undernutrition and obesity that are becoming increasingly connected, according to a four-paper report published in *The Lancet* in December 2019 co-authored by WHO researchers.

More than a third of low- and middle-income countries have populations with both undernutrition and obesity. These overlapping forms of malnutrition were found in 45 out of 123 countries in the 1990s and in 48 of 126 countries in the 2010s.

Undernutrition and obesity can lead to effects across generations as both maternal undernutrition and obesity are associated with poor health in offspring. However, because of the speed of change in food systems, more people are being exposed to both forms of malnutrition at different points in their lifetimes, which compounds the harmful health effects.

“We are facing a new nutrition reality,” said WHO author Dr Francesco Branca, Director of the Department of Nutrition for Health and Development at WHO headquarters in Geneva. “We can no longer characterize countries as low income and undernourished, or high income and only concerned with obesity.

“All forms of malnutrition have a common denominator: food systems that fail to provide all people with healthy, safe, affordable and sustainable diets. Changing this will require action across food systems – from production and processing, through trade and distribution, pricing, marketing, and labelling, to consumption and waste. All relevant policies and investments must be radically re-examined,” Branca said.

The new report explores the trends behind this intersection – known as the double burden of malnutrition – as well as the societal and food system changes that may be causing this problem, the biological explanation and effects, and policy measures that may help address malnutrition in all its forms. 

http://bit.ly/2NtWsFJ

**Cover Photo**

Health workers walking to measles and rubella vaccination sessions in Arunachal Pradesh, India.

**Figure:**
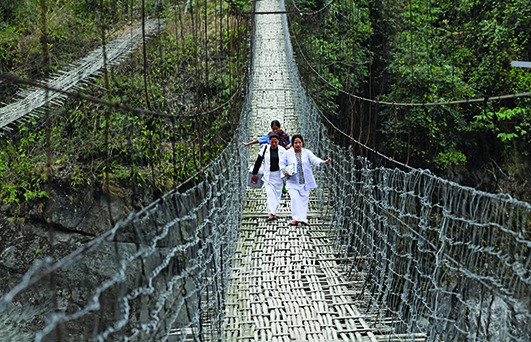
UNICEF/ Biju Boro

## Breast cancer biosimilar prequalified

WHO announced on 18 December that it had prequalified a biosimilar medicine for the first time, breast cancer drug trastuzumab, in a move that could make this expensive, life-saving treatment more affordable and available to women globally.

Breast cancer is the most common form of cancer in women. About 2.1 million women were diagnosed with breast cancer in 2018, of which 630 000 died from the disease, in many cases because of late diagnosis and lack of access to affordable treatment.

Trastuzumab – a monoclonal antibody – was included in the *WHO Model list of essential medicines* in 2015 as an essential treatment for about 20% of breast cancers. It has shown high efficacy in curing early stage breast cancer and, in some cases, more advanced forms of the disease.

The annual cost of trastuzumab from originator companies can be as high as US$ 20 000. The biosimilar version of trastuzumab is generally 65% cheaper than the originator. With this WHO listing, and more products expected in the prequalification pipeline, treatment prices should decrease even further.

The biosimilar medicine, supplied by Samsung Bioepis NL B.V. (Netherlands), was assessed by WHO and found comparable to the originator product in terms of efficacy, safety and quality. That means it is eligible for procurement by United Nations agencies and for national tenders.

Biotherapeutic medicines, which are produced from biological sources, such as cells rather than synthesized chemicals, are important treatments for some cancers and other noncommunicable diseases.

Biosimilars, like generic medicines, can be much less expensive versions of innovator biotherapeutics, while keeping the same effectiveness and are usually manufactured by other companies once the patent on the original product has expired. 

http://bit.ly/2TpAeIN

## Pre-exposure prophylaxis against HIV

People who are taking antiretroviral drugs to protect themselves from acquiring HIV infection because they are considered to be at high risk of HIV infection are also at high risk of other sexually transmitted infections.

Pre-exposure prophylaxis (PrEP) is given to HIV-negative people with a higher-than-average risk of contracting HIV, such as men who have sex with men and people who inject drugs, sero-discordant couples, and young adolescents in certain parts of the world. 

These services could be an ideal place to test for, prevent and treat HIV, as well as other sexually transmitted infections, according to a global study led by Monash University in Australia and supported by WHO that was published in December 2019.

The Melbourne Sexual Health Centre and WHO worked with a team of researchers to conduct a global systematic review evaluating the prevalence and incidence of sexually transmitted infections among individuals using PrEP.

The review, published in *JAMA Network Open*, highlighted the limited focus and investment in the management of sexually transmitted infections within HIV programmes.

Since 2015, WHO has recommended PrEP for people at substantial HIV risk. It consists of a combination of tenofovir and emtricitabine. 

http://bit.ly/38786yk

**Looking ahead**

3 – 8 February. WHO Executive Board Meeting, WHO headquarters, Geneva, Switzerland.

24 – 26 February. First Global Technical Partnership Meeting on Antimicrobial Stewardship, Bangkok, Thailand.

